# Exploring the underlying biology of cancer and potential therapeutic strategies: a special issue focused on mechanism-based studies

**DOI:** 10.3724/abbs.2023114

**Published:** 2023-06-19

Cancer, commonly referred to as malignant tumors, is one of the deadliest human diseases. Every year, millions of people die from cancer, and almost as many are newly diagnosed with the disease. Due to its severe impact on both individuals and the economy, cancer research has become one of the most active areas of biomedical research in recent decades. As cancer originates from highly proliferative, invasive, and immune-suppressive transformed cells, understanding the molecular mechanisms that determine the malignant phenotype of cancer cells is crucial for the development of effective cancer therapy. This special issue comprises 10 reviews by experts in basic cancer research and aims at summarizing the up-to-date advances in cancer biology from their own perspectives.

Signaling pathways are the primary link connecting the extracellular microenvironment and intrinsic cell cycle controlling machinery. Dysregulation of the Hippo pathway, an evolutionarily conserved, but relatively newly identified signaling pathway regulating cell proliferation, has been implicated in multiple cancer types. In two reviews by Cao *et al*. [1] and Zhu *et al*. [2], recent reports on the general signaling network of the Hippo pathway in tumorigenesis are summarized, with a particular in-depth focus on the tumorigenesis and progression of gastric cancer. They also discuss translational implications of targeting the Hippo pathway for cancer treatment.

Aberrant metabolism reprogramming provides energy and building blocks for biomass synthesis to drive cancer progression, enabling cancer cells to proliferate and cope with unfavorable conditions such as hypoxia, oxidative stress during metastasis, and chemotherapy. Altered cancer cell metabolism can release certain metabolites into the microenvironment and cause complex consequences beyond solely affecting cancer cells themselves. Notably, different types and stages of cancers often have distinctive metabolic features, driven by various genetic mutations and environmental pressures. Jiang and colleagues [3] summarize recent knowledge about the role of *TP53*, the most famous tumor suppressor and frequently mutated gene in the human genome, in mediating anabolic and catabolic metabolism in cancer. They also highlight how metabolites regulate *TP53* function. Different from *TP53, KRAS* is one of the most commonly mutated driver genes in human cancers with the highest mutation rate (over 95%) in pancreatic ductal adenocarcinoma (PDAC), which is an extremely lethal cancer type with no effective cure currently available. In another review by Guo *et al*. [4], the mechanisms underlying lipid metabolism reprogramming in PDAC cells and their impact on the microenvironment are introduced, offering new insights into potential therapeutic strategies for PDAC. Metabolic reprogramming not only promotes cancer progression, but also causes therapeutic resistance. Yue *et al*. [5] outline recent studies about the metabolic crosstalk between ovarian cancer cells and the tumor microenvironment (TME), highlighting the role of metabolic reprogramming in mediating the failure of anti-angiogenic therapy in some ovarian cancer patients. Overall, these studies indicate the promising potential of metabolism-targeting strategies in cancer therapy.

Even within the same cancer type, there can be distinct biological features, resulting in differential responses to a particular therapeutic strategy. Tumor heterogeneity can be determined by the mutational landscape of each individual tumor or the TME. Small cell lung cancer (SCLC), the most lethal type of lung cancer, is typically diagnosed at relative late stages, and patients often develop therapeutic resistance shortly after chemotherapy and radiotherapy that are initially effective, leading to relapse and distant metastases. Ji and colleagues [6] summarize the recent understanding of SCLC heterogeneity related to metastases and chemotherapy resistance, providing clues for better intervention strategies. From a different angle, Ren and colleagues [7] focus on recent advances in prostate cancer (PCa) TME study, particularly reviewing PCa TME components and up-to-date TME-targeted therapies, which can provide insights into PCa heterogeneity and therapeutic decisions. Unlike solid tumors, leukemia cells have a unique TME since they originate from the hematopoietic system and are more exposed to immune cells and cytokines in the blood. Mu *et al*. [8] summarize advances in leukemia immunotherapy, using acute myeloid leukemia (AML) and acute lymphoblastic leukemia (ALL) as examples. They discuss bispecific antibody drugs, cell therapies, and checkpoint blockade agents, indicating that effective therapeutic plans may be possible based on a full understanding of cancer heterogeneity and TME.

Covalent RNA modification is a relatively new epigenetic modulation that can affect RNA stability, translation efficiency, and recruitment of interacting proteins/RNA. RNA methylation is the most common and well-studied covalent RNA modification that exerts extensive influence on physiological and pathological activities. In their review, Meng *et al*. [9] overview the regulation, expression patterns, and clinical implications of the three major RNA methylations of m6A, m5C, and m7G in esophageal cancer, and also discuss direct consequences of RNA methylation such as RNA turnover and protein translation, as well as indirect impacts on cancer cells including signaling pathways and the TME. On the other hand, aging is an independent risk factor for cancer. It is widely accepted that the risk of cancer increases in elderly people due to the accumulation of gene mutations over time. However, aging may also affect cancer occurrence by many other mechanisms in addition to causing genome instability. Lin *et al*. [10] focus on the impact of aging on breast diseases, particularly breast cancer, using the breast as an example organ. They introduce the changes in hormonal level, morphology of the terminal duct lobular units, and proportions of adipose tissue that may all contribute to the increased risk of breast cancer during aging, raising the interesting topic of cancer prevention.
